# Therapeutic monoclonal antibody treatment protects nonhuman primates from severe Venezuelan equine encephalitis virus disease after aerosol exposure

**DOI:** 10.1371/journal.ppat.1008157

**Published:** 2019-12-02

**Authors:** Crystal W. Burke, Jeffery W. Froude, Franco Rossi, Charles E. White, Crystal L. Moyer, Jane Ennis, M. Louise Pitt, Stephen Streatfield, R. Mark Jones, Konstantin Musiychuk, Jukka Kervinen, Larry Zeitlin, Vidadi Yusibov, Pamela J. Glass

**Affiliations:** 1 Virology Division, US Army Medical Research Institute of Infectious Disease, Fort Detrick, Maryland, United States of America; 2 Center of Aerobiological Sciences, US Army Medical Research Institute of Infectious Disease, Fort Detrick, Maryland, United States of America; 3 Biostatisics Branch, US Army Medical Research Institute of Infectious Disease, Fort Detrick Maryland, United States of America; 4 Mapp Biopharmaceutical, Inc., San Diego, California, United States of America; 5 Fraunhofer USA Center for Molecular Biotechnology, Newark, Delaware, United States of America; Emory University, UNITED STATES

## Abstract

There are no FDA licensed vaccines or therapeutics for Venezuelan equine encephalitis virus (VEEV) which causes a debilitating acute febrile illness in humans that can progress to encephalitis. Previous studies demonstrated that murine and macaque monoclonal antibodies (mAbs) provide prophylactic and therapeutic efficacy against VEEV peripheral and aerosol challenge in mice. Additionally, humanized versions of two neutralizing mAbs specific for the E2 glycoprotein, 1A3B-7 and 1A4A-1, administered singly protected mice against aerosolized VEEV. However, no studies have demonstrated protection in nonhuman primate (NHP) models of VEEV infection. Here, we evaluated a chimeric antibody 1A3B-7 (c1A3B-7) containing mouse variable regions on a human IgG framework and a humanized antibody 1A4A-1 containing a serum half-life extension modification (Hu-1A4A-1-YTE) for their post-exposure efficacy in NHPs exposed to aerosolized VEEV. Approximately 24 hours after exposure, NHPs were administered a single bolus intravenous mAb. Control NHPs had typical biomarkers of VEEV infection including measurable viremia, fever, and lymphopenia. In contrast, c1A3B-7 treated NHPs had significant reductions in viremia and lymphopenia and on average approximately 50% reduction in fever. Although not statistically significant, Hu-1A4A-1-YTE administration did result in reductions in viremia and fever duration. Delay of treatment with c1A3B-7 to 48 hours post-exposure still provided NHPs protection from severe VEE disease through reductions in viremia and fever. These results demonstrate that post-exposure administration of c1A3B-7 protected macaques from development of severe VEE disease even when administered 48 hours following aerosol exposure and describe the first evaluations of VEEV-specific mAbs for post-exposure prophylactic use in NHPs. Viral mutations were identified in one NHP after c1A3B-7 treatment administered 24 hrs after virus exposure. This suggests that a cocktail-based therapy, or an alternative mAb against an epitope that cannot mutate without resulting in loss of viral fitness may be necessary for a highly effective therapeutic.

## Author summary

Endemic in the Americas, Venezuelan equine encephalitis virus (VEEV) can be transmitted to humans, horses, and other animals through the bite of a mosquito. Beyond its natural prevalence, VEEV was previously developed as a biological weapon making the development of vaccines and therapeutics of the upmost importance. Despite over 60 years of research to identify effective therapeutics for VEEV disease, to-date no anti-VEEV therapeutics have progressed beyond pre-clinical testing in a mouse model. Here, we present the first evaluation of an anti-VEEV therapeutic in a nonhuman primate (NHP). We found that a monoclonal antibody given either one or two days after an aerosol exposure to VEEV protected from severe VEE disease. We also found the level of *in vitro* virus neutralization by a given antibody did not predict efficacy in NHPs. Importantly, we identified viral escape mutations in one NHP after treatment, highlighting the need for development of novel antibodies for inclusion in cocktail-based therapy against VEEV.

## Introduction

An enveloped, single-stranded RNA virus of the *Togaviridae* family, Venezuelan equine encephalitis virus (VEEV), is one of the most extensively studied alphaviruses due to its historical production as a biological agent by multiple State actors [1]. In humans, the virus is rarely lethal, causing a debilitating acute febrile illness which can lead to encephalitis. Despite decades of research, currently no FDA-approved vaccines or therapeutics exist for protection of humans against VEE disease.

The production of neutralizing antibodies against encephalitic alphaviruses following immunization has been a hallmark of protection for decades [2–7]. Numerous studies have demonstrated that administration of neutralizing antibodies, both pre- and post-exposure, can elicit partial or full protection against a peripheral or aerosol VEEV challenge of mice [8–15]. Two particular antibodies identified in 1985, 1A3B-7 and 1A4A-1 [16], have been extensively studied for their protection of mice from both peripheral or aerosol exposure with VEEV [8, 9, 13, 14, 17]. *In vitro*, murine 1A4A-1 had higher binding and neutralization capabilities but less cross-reactivity against VEEV subtypes when compared to murine 1A3B-7 [14, 16]. *In vivo*, a single administration of humanized (Hu)-1A4A-1 provided complete protection of mice from a peripheral challenge when given 24 hours (hrs), but not 72 hrs after infection [9]. Similarly, a single dose of Hu-1A3B-7 administered 24 hrs or 48 hrs after infection protected 70% or 40% of mice, respectively, from homologous aerosol challenge, while no protection was observed when administered 72 hrs after exposure [13]. Further, cross-protection against heterologous strains was demonstrated with ≥80% of mice protected against VEEV complex subtypes IE, II or IIIA [13].

To date, no therapeutic has been reported against VEEV, or any other encephalitic alphavirus in an NHP model. Furthermore, as a biological select agent, VEEV-specific therapeutics will likely require evaluation under the FDA “Animal Rule” where human efficacy studies are neither ethical nor feasible. The cynomolgus macaque has been previously used in VEEV vaccine efficacy studies [18, 19] and displays signs of disease similar to those reported from accidental laboratory exposure [20, 21] suggesting the species is an adequate animal model to demonstrate therapeutic efficacy. In those efficacy studies, onset of symptoms occurs as early as 24 hrs after virus exposure.

Here, we have built upon *in vitro* and murine *in vivo* data by demonstrating efficacy of a murine-human chimeric Ab c1A3B-7 and a humanized Ab Hu-1A4A-1 with a half-life extension modification (YTE) in NHPs and evidence for the continued advancement of antibody-based therapies against VEE into clinical testing. In agreement with previous studies [16, 22], we found that Hu-1A4A-1-YTE had greater neutralizing capacity than c1A3B-7 which has a higher binding affinity and ELISA titer. Despite this, c1A3B-7 was more efficacious in NHPs than Hu-1A4A-1-YTE when administered 24 hrs after aerosol exposure. Further, treatment with c1A3B-7 could be delayed 48 hrs after exposure and still provide protection from severe VEE disease. Our results demonstrate for the first time that a therapeutic mAb can offer post-exposure protection against an aerosolized alphavirus and highlight the opportunity for further mAb development as a medical intervention against this family of viruses.

## Results

### In vitro assessment of chimeric and humanized antibodies

**Fig 1:**
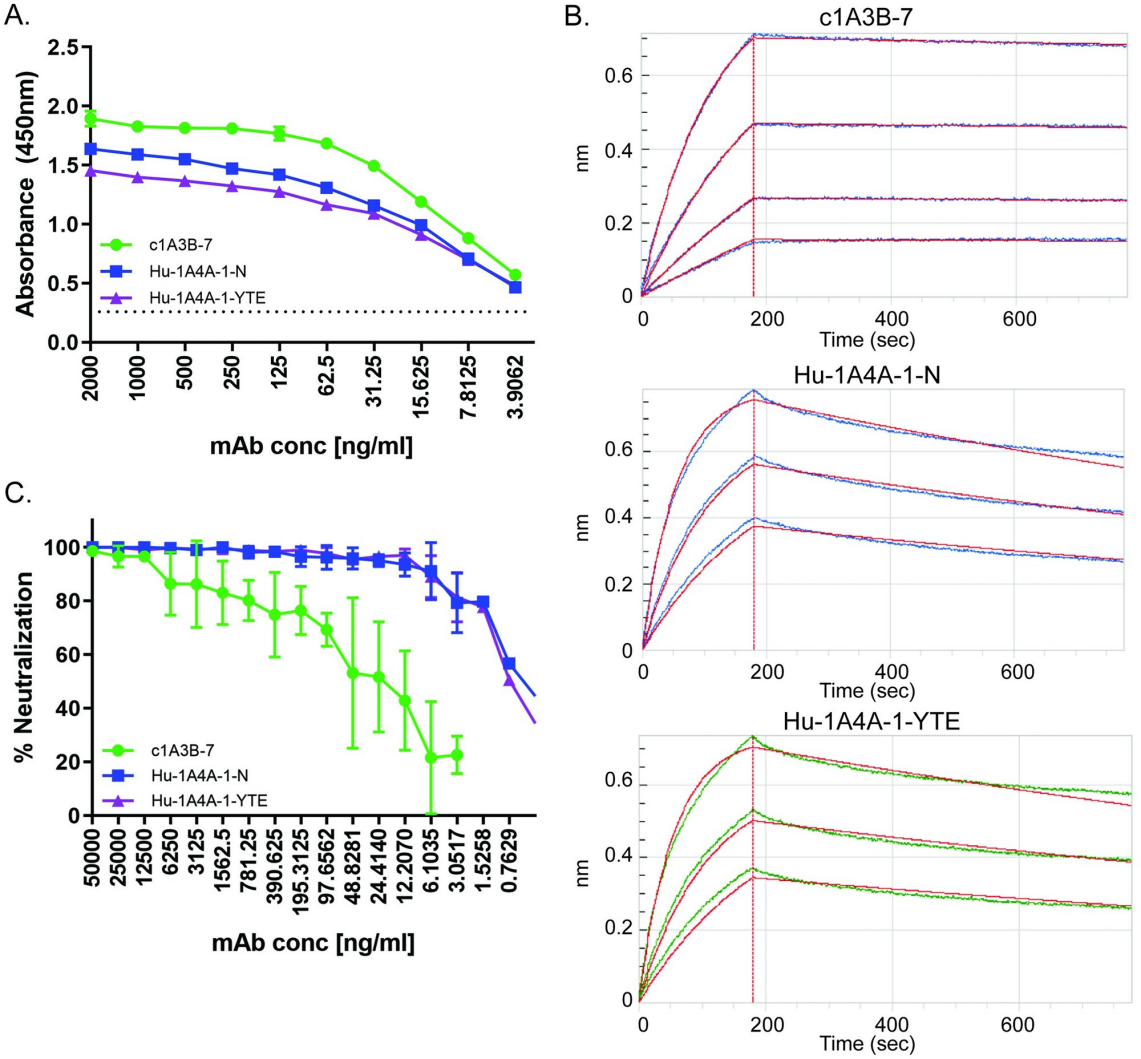
*In vitro* characterization of plant-derived antibodies, c1A3B-7, Hu-1A4A-1-N, and Hu-1A4A-1-YTE. (**A**) ELISAs were performed to determine binding capacity to plates coated with VEEV TrD virus. Equal protein concentrations were used for each mAb. Experiment was performed in triplicate, with duplicate samples. Data shown is representative dataset with SD (most error bars are too small to see). Dotted line represents the limit of detection. (**B**) Kinetic analysis of mAb binding was performed using biolayer interferometry kinetic analysis. Equal protein concentrations (25 μg/ml) of each mAb were used to calculate the K_D_, K_on_, and K_off_ values (Table 1). (**C**) Neutralization was measured using PRNT assay. Equal protein concentrations of each mAb were tested for their ability to neutralize VEEV TrD virus. Experiment was performed in triplicate, with duplicate samples. Points represent the mean data from all experiments with SD.

While small-scale production of both c1A3B-7 and Hu-1A4A-1 was achievable in CHO cells and *N*. *benthamiana* plants, in order to test these antibodies in a NHP model they were scaled up in *N*. *benthamiana* plants. An alternative signal sequence to direct antibodies to the cell surface was utilized in the plant system to allow for efficient, high quality production. The binding capacity and neutralizing activity of the plant-produced c1A3B-7 and an unmodified Hu-1A4A-1-N were compared using VEEV Trinidad donkey (TrD) strain (Fig 1). At equal protein concentrations, c1A3B-7 demonstrated improved ELISA binding compared to Hu-1A4A-1-N and a half-life extension modified variant, Hu-1A4A-1-YTE (Fig 1A) [23, 24]. c1A3B-7 had an overall K_D_ 5-fold greater than the Hu-1A4A-1 Abs by bio-layer interferometry (BLI; Fig 1B and Table 1). However, the Hu-1A4A-1 Abs neutralized >90% of virus by PRNT at concentrations as low as 6 ng/mL while at the same concentration c1A3B-7 neutralized only ~20% of virus (Fig 1C). Comparison of PRNT_80_ and PRNT_50_ values reveals that c1A3B-7 requires 235-fold or 46-fold more protein than the Hu-1A4A-1 Abs to achieve similar levels of neutralization *in vitro* (Table 1). Since the half-life extension modified antibody performed as well *in vitro* as the parent Hu-1A4A-1-N mAb, the Hu-1A4A-1-YTE antibody was used in subsequent studies.

**Table 1:** *In vitro* neutralization and kinetic analysis of anti-VEEV mAb binding to VEEV envelope monomers.

Sample ID	PRNT_80_/PRNT_50_ (ng/ml)^a^	KD (M)	KD Error	K_on_(1/Ms)	K_dis_(1/s)	Full X^2	Full R^2
Hu-1A4A1-YTE	2.408 / 0.675	1.35E-09	1.76E-11	3.20E+05	4.32E-04	0.570	0.990
Hu-1A4A1-N	2.476 / 0.481	1.65E-09	1.60E-11	3.20E+05	5.27E-04	0.442	0.993
c1A3B-7	564.043 / 31.537	3.12E-10	6.90E-12	1.36E+05	4.25E-05	0.045	0.999

^a^PRNT values were derived using a Nonlin fit log(inhibitor) vs. normalized response with variable slope analysis in Graph Pad Prism

The half-life of chimeric and human(ized) mAbs administered to NHP by the intravenous (IV) route is varied in the literature, but is generally between 7–11 days [25]. In a pharmacokinetic study, IM and IV routes of administration were compared for both Hu-1A4A-1-YTE and c1A3B-7. When administered by the IV route, the half-life values for c1A3B-7 and Hu-1A4A-YTE were 7.41 and 22.2 days, respectively.

### Nonhuman primate efficacy study

To establish the utility of a post-exposure mAb-based treatment as a therapeutic for use in humans, we examined the protective efficacy of c1A3B-7 and Hu-1A4A-1-YTE in cynomolgus macaques. Based on the limited human data available, aerosol exposure of cynomolgus macaques to VEEV closely resembles clinical signs observed during accidental laboratory exposures [20, 21]. Groups of adult macaques were exposed to a target inhaled dose of 1.0x10^5^ PFU VEEV TrD in Experiment 1 (calculated average inhaled dose 9.01x10^4^ PFU) and a target inhaled dose of 1.0x10^6^ PFU VEEV TrD in Experiment 2 (calculated average inhaled dose 2.71x10^6^ PFU) by the aerosol route. The target inhaled exposure dose was intentionally increased by a log in Experiment 2 based on the results from Experiment 1. Twenty-four hours (±1 hr) after exposure, cohorts were treated IV with 25 mg/kg c1A3B-7 (Experiment 1; n = 6), Hu-1A4A-1-YTE (Experiment 2; n = 5) or equal volume by weight vehicle control (Experiment 1, n = 6; Experiment 2, n = 5). Blood was collected daily through day 7 and weekly thereafter until day 28. Based on markers of VEEV disease defined in NHP vaccine studies [18, 19], viremia and temperature were used as the primary efficacy endpoints for these initial proof-of-concept studies. The secondary efficacy endpoints assessed were lymphopenia and neutropenia, defined as a ≥ 30% reduction in absolute lymphocyte or neutrophil values compared to pre-exposure levels (Table 2).

**Table 2:** mAb-induced protection and clinical observations in NHPs aerosol exposed to VEEV.

	Treatment Group	# of NHPs (N)	Total Viremia^a^ Median (QR)	Temperature			Lymphopenia^e^		Neutropenia^f^	
				T-Max Mean^b^ (SD)	Fever Duration Mean^c^ (SD)	Fever-hours Mean^d^ (SD)	Mean # Days (SD)	Median % change in ABS lymphocytes (QR)	Mean # Days (SD)	Median % change in ABS neutrophils (QR)
**Exp 1**	**Control**	**6**	23300 (7200)	2.1 (1.07)	32.8 (33.35)	172.6 (147.20)	4.2 (0.75)	-36.6 (8.39)	3.5 (1.52)	-33.9 (32.80)
	**c1A3B-7** **(DOC+1)**	**6**	154* (7080)	1.9 (0.71)	15 (15.39)	116.2 (81.77)	1.8† (1.47)	-18.4* (24.60)	1.5† (2.07)	11.3* (30.00)
**Exp 2**	**Control**	**5**	1130 (3570)	2.8 (0.96)	74.3 (49.77)	324.1 (189.40)	3 (1.41)	-26.6 (3.70)	3.2 (1.30)	-9.04 (24.30)
	**Hu-1A4A-1-YTE** **(DOC+1)**	**5**	8250 (9290)	2.4 (0.76)	60 (42.39)	215.4 (145.20)	1.8 (1.79)	-23.9 (3.28)	3.6 (2.07)	-25.2 (25.60)
	**c1A3B-7** **(DOC+2)**	**5**	1230 (1180)	2.4 (0.81)	40.6 (27.06)	145.1 (84.18)	2 (1.22)	-16.2 (11.30)	2.4 (1.52)	-5.6 (28.40)

*p<0.05 Wilcoxon Exact Test vs. Control

^†^ p<0.01 Logistic Regression Test

^a^PFU/mL

^b^The group mean of the maximum residual temperature elevation in degree Celsius

^c^Average number of 30 minute intervals with temperature >1°C above baseline across 28 days ÷ 2; must have 2 consecutive events to be counted (1 full hour >1°C)

^d^The group mean of the sum of significant temperature elevations (>3SD above baseline) in Celsius-hours

^e^Lymphopenia defined as a >30% reduction in absolute (ABS) lymphocyte counts compared to the average of 3 baseline values

^f^Neutropenia defined as a >30% reduction in absolute (ABS) neutrophil counts compared to the average of 3 baseline values

SD = Standard Deviation, QR = Quantile Range, DOC = Day of Challenge

**Fig 2:**
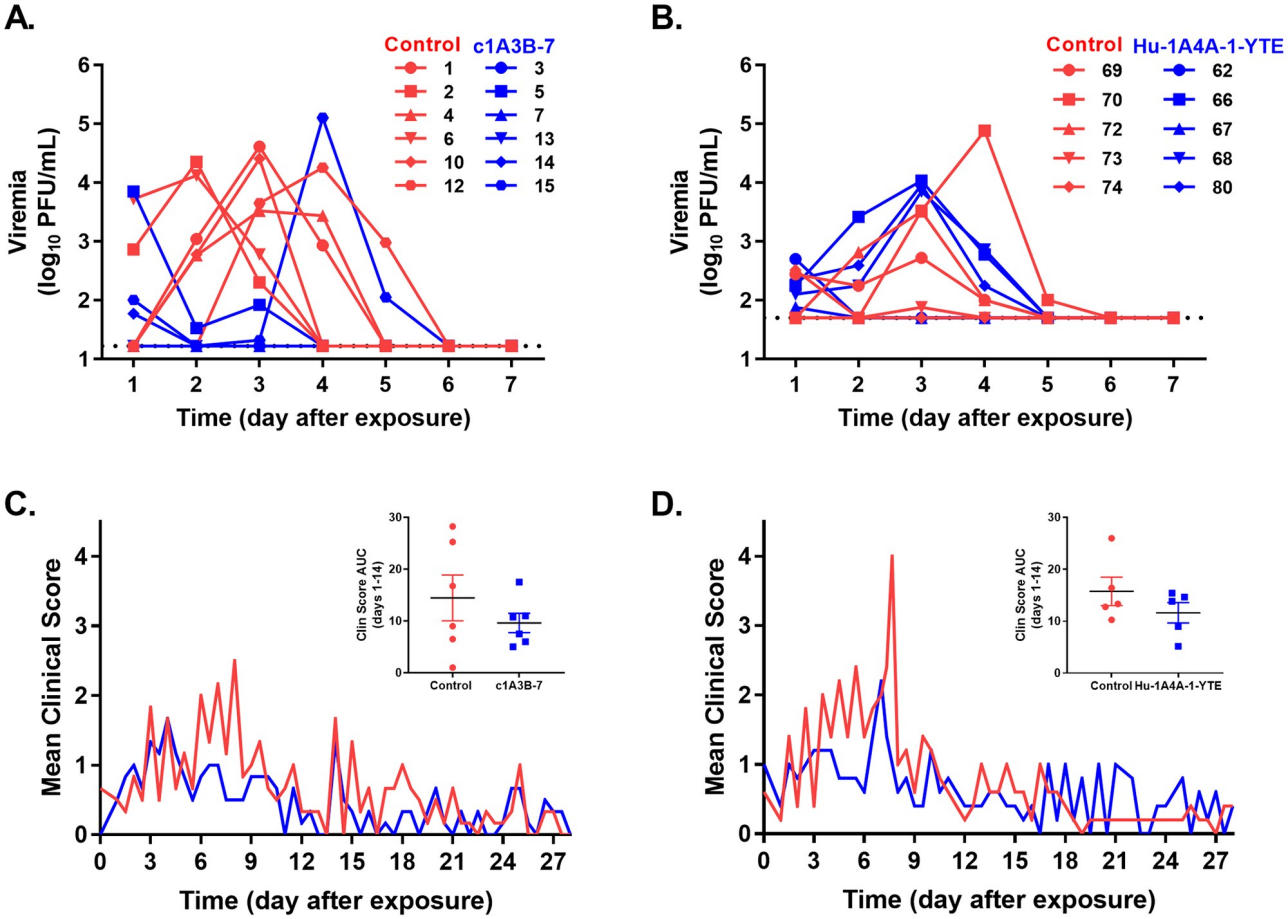
Viremia and clinical scores in mAb or PBS treated (control) macaques after VEEV aerosol exposure. Blood was collected on days 1–7 after aerosol exposure and the levels of infectious virus were measured by plaque assay. (**A**) NHPs were administered c1A3B-7 or PBS on24 hrs after virus exposure. Infectious virus was detected in blood by plaque assay. Following administration of treatment, viremia was absent in 4 of 6 mAb-treated NHPs compared control NHPs which were viremic for up to 5 days after exposure. Each line represents an individual NHP. Dotted line represents the limit of detection. (**B**) NHPs were administered Hu1A4A-1-YTE or PBS 24 hrs after virus exposure. Hu1A4A-1-YTE treated NHPs had similar amounts of virus in the blood as control NHPs. Each line represents an individual NHP. Dotted line represents the limit of detection. (**C**) c1A3B-7 or (**D**) Hu1A4A-1-YTE treatment reduced the mean clinical scores of the NHPs when compared to control animals. These reductions were more apparent when comparing the area under the curve for individual animals between days 1–14 (inset). Individual data points represent each NHP, line represents mean, and error bars are SEM.

Although the magnitude varied amongst the control cohort in each study, all NHPs administered vehicle control had measurable viremia (Fig 2, panels A and B). Protection from viremia was observed in NHPs administered c1A3B-7 as only 2 of the 6 NHPs had measurable levels of infectious virus in the blood post-treatment (Fig 2A). Hu-1A4A-1-YTE treatment did not protect NHPs from developing viremia, but did result in a reduction of blood virus titers in 2 out of 5 NHPs following mAb administration (Fig 2B). Interestingly, one c1A3B-7 treated NHP had a spike of viremia on day 4 after exposure (Fig 2A, NHP 15). Deep sequencing of this sample identified nucleotide changes in the E2 glycoprotein. These changes may represent viral Ab escape mutations [26]. Future studies will evaluate this possibility. Of note, some control NHPs in the Experiment 2 only had low-levels of virus present in the blood after infection. Due to a slight reduction in viremia and fever in controls of Experiment 1, the virus challenge dose was increased from 10^5^ to 10^6^ PFU per NHP; yet even with this increase, several control NHPs had lower viremia. This outcome was unexpected and it is possible that the vehicle control (PBS) administered IV at a dose of 1.2 ml/kg provided a supportive care effect. A similar effect has been noted in mice administered PBS following infection (S1 Fig). This possibility should be further evaluated and will likely need to be taken into consideration in future studies evaluating post-exposure treatments.

VEEV infection of cynomolgus macaques results in a non-lethal disease. Clinical scores collected by blinded study personnel are a daily summation of the overall health of the animal taking into consideration the animal’s temperature, responsiveness, and clinical signs consistent with neurological dysfunction. NHPs administered c1A3B-7 (Fig 2C) or Hu-1A4A-1-YTE (Fig 2D) had overall lower mean clinical scores than NHPs administered PBS, although the range in clinical scores in control NHPs was more variable than observed in mAb-treated NHPs (Fig 2C & 2D inset). This variability in clinical presentation of VEE disease is similar to what is observed in humans [21, 27]. Within the first week after exposure, vehicle control animals exhibited mild signs of disease including anorexia, lethargy, reduced movements, and hunched posture. Severity of disease in controls ranged from no apparent tremors (4/11) to slight action-related tremors in extremities (4/11) to full-body tremors (3/11). Less frequent observations including hyper reactivity to sound or movement (2/11), aggression (2/11) and apparent photophobia (3/11) were also noted. NHPs administered c1A3B-7 displayed subdued or depressed behavior with some animals having slight action-related tremors (4/6) and one animal having apparent photophobia and hyper reactivity. Subdued behavior, some anorexia (1/5) and slight action tremors (4/5) were noted in Hu-1A4A-1-YTE treated NHPs.

**Fig 3:**
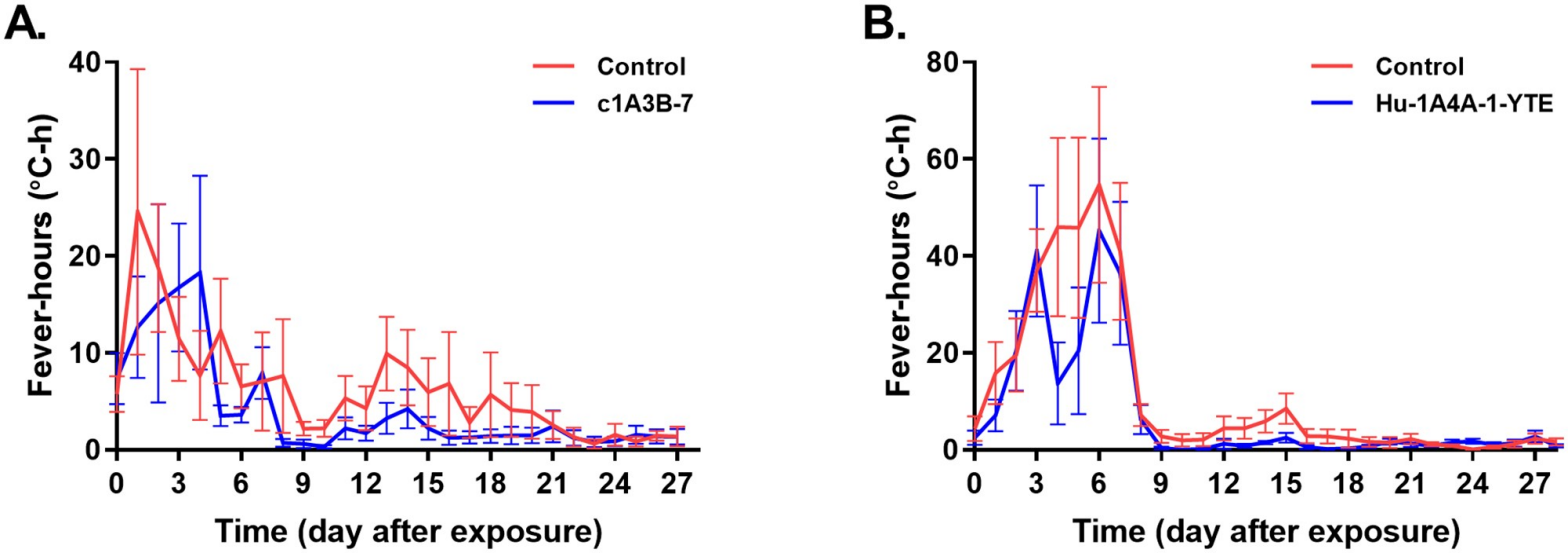
Temperature comparisons between control and mAb-treated NHPs. The temperature of individual NHPs was collected constantly via implanted telemetry devices. Baseline data was collected to define the normal circadian patterns for each NHP. After VEEV exposure, temperatures were compared to baseline values to determine changes and calculate fever-hours. Administration of c1A3B-7 (**A**) and Hu1A4A-1-YTE (**B**) 24 hrs after virus exposure decreased the average fever-hours over the course of the study when compared to the control cohort. Data are displayed as the mean, error bars represent standard error of the mean (SEM).

To monitor temperature, NHPs were implanted with telemetry devices. Temperature data for each animal were continuously collected for at least 5 days prior to aerosol exposure and were used to determine the baseline values for post-exposure temperature monitoring. Fever-hours were calculated for each NHP and average fever-hours per treatment group were compared (Fig 3A & 3B). Defined as the sum of the hourly temperature elevation values greater than 3 standard deviations (SD) above baseline for a 24 hour time period, fever-hours gives an indication of the intensity of the temperature elevation by measuring the area under the curve. On average, control NHPs in both studies had greater instances of elevated temperatures over the course of the study when compared to the mAb-treated animals, although treatment with the mAb did not eliminate increase in temperatures. Additional comparisons were made between maximum temperature values (TMax) and fever duration, defined as the number of hours (60 consecutive minutes) of temperature elevated > 1 degree Celsius above baseline. No significant changes in TMax were observed across all treatment groups (Table 2). On average, c1A3B-7 treated NHPs experienced a ~50% reduction in fever duration while Hu-1A4A-1-YTE treatment reduced fever duration by <20%.

A hallmark of VEE disease in humans [27, 28] and NHPs [18, 19], lymphopenia was used as a secondary efficacy endpoint. Complete blood cell counts were performed daily for the first week after exposure and compared to baseline values. Characteristically, control NHPs had drastic decreases (>30%) in absolute lymphocyte counts observed during the first week lasting on average 3 to 4.2 days depending on the study (Table 2). NHPs receiving c1A3B-7 mAb experienced both a reduction in the number of days of lymphopenia (average 1.8 days) as well as a significant reduction in the median % change in absolute lymphocyte values (-18.4 vs. control -36.6; p<0.05). Hu-1A4A-1-YTE administration reduced the average number of days of lymphopenia as well (1.8 days vs. control 3 days) although only minor differences were observed between the median % changes in absolute lymphocytes. Similar comparisons were made in absolute neutrophil counts as neutropenia has been observed in humans [28, 29]. Animals administered c1A3B-7 had a reduction in the mean total number of days of neutropenia (1.5 days vs. control 3.5 days) and the median % change in absolute neutrophils (11.3 vs. control -33.9; p<0.05). No significant changes in neutropenia were observed with administration of Hu-1A4A-1-YTE.

### Delayed administration of c1A3B-7 treatment

**Fig 4:**
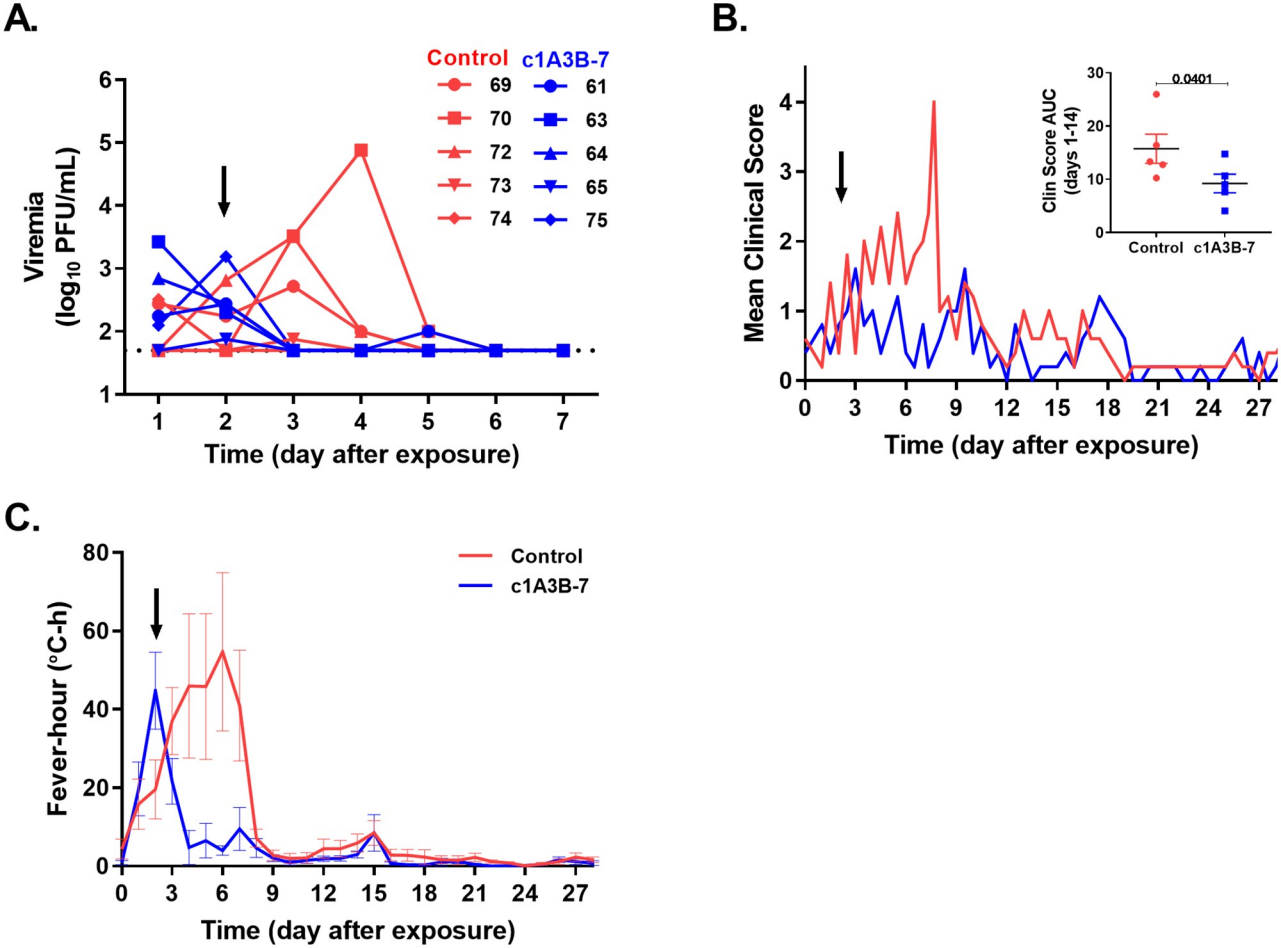
Efficacy of c1A3B-7 when administered 48 hrs post-exposure. NHPs were aerosol exposed to VEEV TrD and c1A3B-7 was administered 48 hrs after exposure. Blood was collected on days 1–7 after VEEV TrD aerosol exposure. NHPs were monitored twice daily for clinical signs of disease. (**A**) Infectious virus was detected in blood by plaque assay. After c1A3B-7 administration infectious virus was reduced to undetectable levels in all animals with a rise above this level in 1 of 5 NHPs on day 5 post-exposure. Dotted line represents the limit of detection. (**B**) Clinical scores in all NHPs were similar prior to mAb administration; however, after administration c1A3B-7 treated NHPs had lower clinical scores which were significantly lower over the acute phase of disease (inset- AUC days 1–14; p = 0.0401 unpaired, one-sided t-test). For inset, individual data points represent each NHP, line represents mean, and error bars are SEM. (**C**) A drastic reduction in fever-hours for the c1A3B-7 treated NHPs was observed. Data are displayed as the mean, error bars represent SEM. Arrows indicate time of c1A3B-7 administration.

Since reductions in the hallmarks of VEE disease (viremia, fever, and lymphopenia) were observed when c1A3B-7 was administered 24 hrs after exposure, we examined whether delaying treatment to 48 hrs after exposure would still be effective. Groups of cynomolgus macaques (n = 5) were exposed to a target inhaled dose of 1.0x10^6^ PFU VEEV TrD (calculated average inhaled dose 2.71x10^6^ PFU) by the aerosol route. To reduce control animal numbers, administration of c1A3B-7 48 hrs after exposure was examined as part of Experiment 2 described above. As such, it is important to note that the control cohort received vehicle control at 24 hrs after exposure, which may have altered total viremia profiles. Total viremia comparisons following Ab administration showed trends in reduction although not statistically significant (two-tailed Wilcoxon test p value 0.0625). However, viremia was detected in 5/5 NHPs prior to c1A3B-7 administration and 24 hrs after treatment virus was no longer detected in all but one NHP which had levels just above the limit of detection (Fig 4A). Early clinical scores were similar between treated and control cohorts; however, after administration of c1A3B-7, NHPs displayed significant reductions in clinical scores during the acute phase (Fig 4B; p = 0.04). Treatment with c1A3B-7 also reduced the fever response by approximately 50% after administration when compared to the control NHPs (Fig 4C & Table 2). However, delayed administration of c1A3B-7 did not significantly alter the magnitude of changes in blood lymphocyte or neutrophil counts when compared to the control NHPs, although the number of days of lymphopenia and neutropenia were reduced (Table 2).

Taken together, the data suggest that c1A3B-7 provided superior protection against a VEEV aerosol exposure in nonhuman primates than Hu-1A4A-1-YTE and was efficacious when administered up to 48 hrs after exposure.

## Discussion

The use of monoclonal antibodies for both prophylactic and therapeutic indications are expanding for a range of infectious diseases [30–36]. Further, anti-VEEV neutralizing antibody levels after vaccination have correlated with protection from VEE disease in mice and NHPs [19, 37–39]. Here, we evaluated two anti-VEEV monoclonal antibodies and demonstrated for the first time in NHPs that post-exposure administration of a mAb could protect against severe VEE disease, even when administered 48 hrs after aerosol exposure. It is important to note, that while Hu-1A4A-1-YTE had a higher neutralizing activity and a longer half-life, its performance *in vivo* was sub-optimal when compared to c1A3B-7 which had a higher binding affinity *in vitro*. A recent study with Western equine encephalitis virus (WEEV)-specific mAbs found that a non-neutralizing mAb was able to protect mice from a lethal aerosol exposure [40]. While screening strategies for novel anti-VEEV mAbs have traditionally focused on neutralization, our data would suggest that characteristics other than neutralization potency may be important for efficacy *in vivo [41]*. The importance of balancing antibody binding kinetics, epitope access, *in vitro* neutralization, mechanism of action, and ability to recruit effector function activity to identify the most effective therapeutic antibodies has been demonstrated with Ebola virus antibody development [42].

In this study, we demonstrated that viremia, lymphopenia, neutropenia, and temperature can be used as efficacy endpoints for pre-clinical VEEV therapeutic studies in nonhuman primates. These parameters have been used previously as indicators of vaccine efficacy [18, 19, 37, 43]. However, in vaccine studies higher virus challenge doses of VEEV (~1x10^8^ PFU) were used resulting in uniform viremia, lymphopenia and temperature elevations in control animals. In an effort to challenge with an Infectious Dose (ID) 99, the targeted inhaled doses were 100–1000 fold lower (target inhaled doses of 1x10^5^ and 1x10^6^ PFU) than previously published vaccine studies. This reduced challenge was chosen based on NHP studies with the VEEV INH-9813 strain, a member of the IC subtype. With this challenge dose, disease parameters in control animals were more variable than observed with higher doses. To increase consistency in therapeutic efficacy endpoints in control animals, ID_50_ studies should be completed for VEEV TrD strain in cynomolgus macaques. Based on our results, we expect that the ID_50_ dose of the TrD strain will be higher than that of the INH-9813 strain; although studies are needed to confirm. Alternatively, ID_50_ of the two strains may be similar, but the administration of diluent may have had a supportive care effect. We and others have noted that administration of PBS after VEEV aerosol challenge of mice may result in some animals surviving an otherwise lethal dose of virus (S1 Fig and CL Gardner, personal communications). These studies highlight the need for additional VEEV model development studies that evaluate the effect of fluid administration post-virus exposure in order to determine the number of animals required to sufficiently power a therapeutic efficacy study, as FDA licensure of any anti-VEEV therapeutic will likely utilize the Animal Rule.

Each disease condition or stage may require a unique therapeutic with varied mechanisms of action. In the case of encephalitic alphaviruses, the ability of the virus to cross the blood brain barrier highlights a particular limitation and challenge for the utilization of traditionally designed mAbs. However, in the absence of any other therapeutic option, having circulating systemic mAbs reduces the overall viral burden while supporting activation of the immune system. In this study, we observed reductions not only in viremia, but in fever, lymphopenia, and neutropenia when c1A3B-7 was administered 24 or 48 hrs after exposure. This successful proof-of-concept utilization of an IgG format mAb is highly supportive of the use of mAbs for the post-exposure treatment of VEE disease. The field of antibody development has been rapidly expanding and the variable regions of protective mAbs to VEEV and other alphaviruses can be reformatted to new constructs. These next generation formats could include, but are not limited to, the ability to cross the blood brain barrier [44].

Lastly, we observed a late spike in viremia on day 4 in one of the c1A3B-7 treated NHPs. This abrupt increase in viral titer is suggestive of viral escape mutant variants. Deep sequencing of serum from that animal identified nucleotide changes in the E2 glycoprotein. 1A3B-7 specifically binds the E2^h^ epitope [16] and others have demonstrated that single amino acid changes at 207 [26, 45] and 209 [45] in the E2^h^ epitope results in a reduction or inhibition of 1A3B-7 binding. Antibody-escape mutants have been identified for other viruses, including Ebola virus [46], influenza virus [47], Dengue virus [48], and other alphaviruses [49, 50]. One possible explanation for the lower therapeutic efficacy of Hu-1A4A-1-YTE is that mutation of the epitope site for this antibody occurs more frequently or rapidly; however, sequencing of samples from NHPs treated with Hu-1A4A-1-YTE would be necessary to further explore this hypothesis. For this reason, it may be important to identify alternative or additional mAbs that could be used in combination with c1A3B-7 to reduce the potential for escape mutants to arise during the course of VEEV disease.

This study highlights for the first time that mAbs can protect NHPs from an encephalitic alphavirus. However, there are some challenges that will need to be addressed prior to taking Ab candidates into the clinic. In addition to the need for a refined infection model, the development of novel mAbs to these encephalitic alphaviruses should be expanded in order to provide additional therapeutic options. RNA viruses have inherently high rates of mutation and may require an antibody cocktail to exploit multiple epitope targets in order to overcome the possibility of escape mutants.

## Materials and methods

### Study design

Study data was collected from two independent, but related, experiments. Experiment 1 compared 25 mg/kg of 1A3B-7 applied one day after challenge to a PBS control. Experiment 2 compared 1A3B-7 (DOA+2) and 1A4A-YTE (DOA+1) to a PBS control. Both of these experiments were intended to be proof-of-concept for mAb protection after VEEV exposure in NHPs. Within experiment, NHPs were randomized to experimental groups and their order of challenge was randomized within experimental group using computer-based random number generation (SAS). Both experiments were originally designed to compare 5 NHPs in each treatment cohort to 5 NHPs in a control cohort. Using a one-sided Fisher’s Exact Test with more than 80% power, five (5) animals/group were expected to detect differences in viremia detection fractions of 0.01 in a treatment group and 0.99 in the control group. Telemetry failures in Experiment 1 resulted in re-randomization and assignment of NHPs into two cohorts with n = 6 prior to challenge. Primary, secondary and additional endpoints are the same for both experiments and were chosen based on human disease [21, 27, 28] and NHP studies [51]. The primary endpoint is total viremia. The secondary endpoint is fever-hours. Additional endpoints include percent lymphocytes and percent neutrophils relative to baseline measurements for individual NHPs. The primary objective is to demonstrate that a treatment provides a reduction in viremia relative to the control (efficacy). The secondary objective is to demonstrate that a treatment provides a reduction in fever (efficacy). Additional objectives include demonstration that one or more treatments increase lymphocytes and neutrophils relative to the control. All data meeting the quality requirements of the measurement methods were included in experimental analyses. Subjects with missing data were not replaced nor were individual missing values within any subject’s record imputed. Missing data were handled as missing at random and no corrections for missing data were included in the analyses. All study personnel performing observations during critical phase of disease were blinded to treatment status.

### Ethics statement

Research was conducted under a USAMRIID IACUC- and USAMRDC ACURO-approved protocol in compliance with the Animal Welfare Act, PHS Policy, and other Federal statutes and regulations relating to animals and experiments involving animals. The facility where this research was conducted is accredited by the Association for Assessment and Accreditation of Laboratory Animal Care, International and adheres to principles stated in the Guide for the Care and Use of Laboratory Animals, National Research Council, 2011. Cynomolgus macaques were of Chinese origin, ≥4 years of age, ≥ 3 kg, alphavirus naïve, and free of specific pathogens. Upon receipt, NHPs were given a physical examination and housed with physical enrichment. NHP rooms were maintained on a 12-hour light/dark cycle with temperature and humidity conditions maintained between 64–84°F and between 30–70% humidity. NHPs were fed primate chow supplemented with dietary enrichment (fruits and vegetables) at least 3 times per week. The amount of primate chow given to each NHP was proportional to their weight. Additional sensory enrichment and occupational enrichment were provided. All NHPs received water through automatic watering systems attached to each cage rack. Following movement into containment, NHPs were also offered flavored Pedialyte in bottles attached to the front of the cage.

### Aerosol generation and calculations

The aerosol challenge dose for each animal was calculated from the minute volume determined with a whole body plethysmograph box using Buxco XA software directly prior to aerosol exposure. The total volume of aerosol breathed was determined by the exposure time required to deliver the estimated inhaled dose. The aerosol challenge was generated using a Collison nebulizer to produce a highly respirable aerosol (flow rate 7.5 ± 0.2 L/minute). The system generated a target aerosol of 1–3 μm mass median aerodynamic diameters determined by TSI Aerodynamic Particle Sizer. Animals were placed in a head-only Automated Bioaerosol Exposure System (ABES-II) System during challenge. Samples were collected from the pre-spray suspension and aerosol collected from the exposure chamber using an all glass impinger (AGI) during each challenge. Samples were stored at -60°C to -90°C until titer was determined by plaque assay. Calculated inhaled dose for each NHP was determined using the following equation: Inhaled Dose (PFU) = [Aerosol] * MV * Run Time, where aerosol concentration is the amount of virus (PFU/mL * volume in AGI sampler /sampler flow rate (mL/min) * sampling duration (min); minute volume (mL/min) and run time (min) is the total time of exposure and air wash.

### Antibodies and administration

1A3B-7 was expressed in *Nicotiana benthamiana* plants and purified over a 3-column (MabSelect, HiTrap SP HP, Capto Q; all resins from GE Healthcare) process. The Hu-1A4A-1-N was modified to include half-life extension substitutions, specifically YTE [23, 24], producing Hu-1A4A-1-YTE. Hu-1A4A-1-YTE was expressed in *Nicotiana benthamiana* plants and purified over a 3-column (MabSelect, Capto Q, CHT) platform process.

Antibodies were administered at 25 mg per kg of NHP body weight. Macaques were weighed within 5 days before challenge for dose calculations. On the day of administration, NHPs were anesthetized with Telazol and mAbs were delivered as a slow push into the saphenous vein.

### Virus stock

The VEEV Trinidad donkey strain was originally isolated from donkey brain in the early 1940’s [52]. Passage history of the virus isolated used in these studies includes 1 pass in guinea pig brain, 14 passes in embryonated chicken eggs, 1 pass in suckling mouse brain, one pass on Vero cells, and one pass on BHK cells. This stock was identified as VEEV Trinidad donkey strain by PCR analysis and deep sequencing. Sterility and mycoplasma test results were negative and endotoxin levels were below the allowable limits.

### Complete blood counts

Blood was collected on three days prior to and within 2 weeks of challenge to establish baseline hematology counts and days 1–7, 14, 21, and 27 or 28 after exposure for alterations in cell counts for each NHP. Whole blood was collected into a pediatric blood tube containing K_2_ EDTA and tubes were gently inverted by hand and on a tube rocker to ensure adequate mixing. Cell counts were measured using a Cell Dyn 3700 clinical hematology analyzer within 2 hrs of collection.

### Telemetry and fever determination

At least 20 days prior to study start, macaques were implanted with telemetry implants (Study 1 ITS-T2J or Study 2 DSI-M00) for collection of temperature and activity data. Approximately 10 days prior to aerosol exposure, NHPs were transferred into the animal biosafety level-3 (ABSL-3) containment facility for acclimation and collection of baseline telemetry data. Temperature data was collected continuously for at least 5 days prior to aerosol exposure at a rate of one sample per second. Data were captured and archived as digital data in proprietary file format (Notocord Structured Storage or NSS) and processed using the Notocord-hem Evolution software platform (version 4.3, NOTOCORD Inc. Newark, NJ 07101, USA). Data were transferred to a GLP validated Excel spreadsheet for analysis. The average of all values corresponding to the same time of day during the baseline period were used to produce a 24-hr period normal baseline reference temperature table for each hour of the day. The standard deviation (SD) was calculated and the significant temperature increase threshold was set for each 30-min interval as greater than the average + 3SD compared the corresponding baseline value. Fever was defined as temperature elevations greater that 1°C above baseline. Following aerosol exposure, NHPs were monitored for 28 days post-exposure. Post-exposure temperatures were used in the calculation of a daily clinical score to describe the overall condition of each NHP. Change in temperature values were calculated by subtracting the average baseline temperature values from the study temperature values of the correspondent daily time period. Study temperature values that were 3SD above or below baseline values were used to generate the daily maximum temperature values (TMax; the highest Δ temperature value for a 24hr daily time period) and daily temperature-hour (temp-hr; the sum of the hourly temperature elevation values for a 24-h time period and gives an indication of the intensity of the fever by crudely measuring the area under the curve). The fever duration were the number of hours of temperature elevation ≥1°C above baseline for two consecutive 30-min intervals within the 28 day study.

### ELISA

High binding 96-well plates were coated overnight at 2–8°C with 5 μg/ml sucrose purified VEEV TrD in PBS. Next, coated plates were fixed with 10% NBF for at least 24 hrs. Fixative was removed, plates were washed 3x with PBS + 0.02% Tween-20 (PBST) and then blocked with PBS + 0.02% Tween-20 + 5% nonfat dry milk + 3% normal goat serum (PBSTMG) overnight at 2–8°C. After blocking, plates were washed three times with PBST. Known protein concentration of samples were added to the plate in duplicate and diluted down the plate in two-fold dilutions in PBSTMG. Plates were incubated for 1–2 hrs at ambient temperature. Following incubation, plates were washed three times with PBST. After washing, goat anti-human IgG (H+L) horseradish peroxidase-conjugated antibody (KPL, Gaithersburg, MD) diluted 1:5,000 in PBSTMG was added and incubated for 1–2 hrs at ambient temperature. After incubation with the secondary antibody, plates were washed three times with PBST, TMB substrate (KPL, Gaithersburg, MD) was added and plates were incubated 3–5 min (time kept constant in each experiment) at ambient temperature at which time the reaction was stopped with 1% HCl. Absorbance was read using a Spectramax M5 instrument set at 450 nm. Assay was performed in triplicate with duplicate samples.

### Kinetic analysis of mAb binding to VEEV antigen

The entire ectodomain (E3/E2/E1) of the VEEV envelope glycoprotein (VEEV ENV) was expressed in Drosophila S2 cells and affinity purified with a StrepTrap HP column (GE Healthcare) via a C-terminal double Strep-Tag. An additional size-exclusion chromatography step on an S200Increase column (GE Healthcare) yielded monomeric ENV antigen. Subsequent biolayer interferometry kinetic analyses were performed with an Octet Red system (ForteBio). Anti-human IgG Fc capture tips were loaded with purified antibodies (25 μg/ml) diluted in kinetics buffer (Gibco PBS pH 7.4 supplemented with 0.002% Tween-20 and 1 mg/ml bovine serum albumin). Following a baseline step, the tips were transferred to wells containing two-fold serial dilutions of VEEV ENV antigen. Kinetic analyses using a 1:1 mode of binding was performed with the ForteBio software to calculate K_D_, K_on_ and K_off_ values as reported in Fig 1.

### Plaque Reduction Neutralization Assay (PRNT)

*In vitro* neutralization was measured using the PRNT. Briefly, antibody samples of known protein concentration were diluted in MEM with 2% heat-inactivated FBS, 1% HEPES, and 2% Pen/Strep and then serially diluted 1:2. Virus stocks were diluted to a concentration of 2.0 x 10^3^ PFU/ml and added 1:1 to the serially diluted samples or control well containing media alone for the virus only control. All samples were incubated overnight at 2–8°C. 6-well plates of Vero 76 cells were grown to ~90–100% confluence. Cells were infected with 0.1 mL of each serial dilution per well in duplicate. Plates were incubated at 37 ± 2°C for 1h ± 15 minutes with gentle rocking every 15 minutes. After 1h, cells were overlaid with 0.6% agarose in Basal Medium Eagle (BME) with 10% HI-FBS, and 2% Pen/Strep, and incubated for 24 ± 4h at 37 ± 2°C, 5 ± 1% CO_2_. A second overlay containing 0.6% agarose in BME with 10% HI-FBS, 2% pen/strep, and 5% of total volume neutral red vital stain (Gibco 02-0066DG) was added to wells and further incubated 18-24h for visualization of plaques. Plaques were counted following incubation with stain overlay. The virus only control was counted and the percent neutralization at each protein concentration was determined. Assay was performed in triplicate with duplicate samples.

### Plaque assay

Plaque assay was used to assess infectious virus in blood. Briefly, samples were diluted in Hank’s Balanced Salt Solution (HBSS), 2% heat-inactivated FBS, 2% Pen/Strep, and 1% HEPES. Vero 76 cells seeded on 6 well plates were grown to ~90–100% confluence. Cells were infected with 0.1 mL of each serial dilution per well in duplicate. Plates were incubated at 37±2°C for 1h ± 15 minutes with gentle rocking every 15 minutes. After 1h, cells were overlaid with BME containing 10% heat-inactivated FBS, 2% Pen/Strep, and 0.6% agarose and incubated 24±4h at 37±2°C, 5±1% CO_2_. A second overlay consisting of BME with 10% heat-inactivated FBS, 2% pen/strep, 5% of total volume neutral red vital stain, and 0.6% agarose was added to wells and further incubated 18-24h for visualization of plaques.

### Statistical analysis

Viremia measurements below detection were set equal to the detection limit (5 PFU/mL). There were no detection limits for lymphocytes and neutrophils. Percent change results were calculated from measurement results while lymphopenia and neutropenia were both calculated from percent change. Baseline for an individual NHP was the average of three pre-challenge measurements, taken on different days. Percent change was then calculated as 100 x [Measurement-Baseline]/Baseline. Presence of lymphopenia was designated as 1 when percent change for lymphocytes was less than or equal to -30% and as 0 when not present. Neutropenia was calculated in a similar fashion. NHP level summary measures [53] were calculated for descriptive statistics and Wilcoxon rank sum tests. For viremia, the summary measure for an NHP was the total of all measurements. In both experiments, there were five (5) viremia measurements per NHP. For percent change, the summary measure for an NHP was the mean of daily measurements for the first seven (7) days after challenge in both experiments. Substituting summary measures for measurements collected over a period of time allowed for meeting the usual assumption of independent measures without explicitly including time in the test. Exact Wilcoxon Rank Sum Tests [54] were used to compare median differences (not the difference in medians) between summary measures for the treatment and associated control groups (S1–S3 Tables). This non-parametric test required few assumptions and it was resistant to the use of detection limits [55]. Exact Logistic Regression was used to calculate odds ratios between treatment and associated control groups [56]. In order to address the assumption that daily measurements on an NHP were treated independently, analysis was conducted on lymphopenia (or neutropenia) values associated with each day and day was included as a factor in the Logistic regression model. Descriptive statistics with the NHP as the fundamental unit of analysis were provided for viremia (S4 Table), lymphocytes, lymphopenia, neutrophils, and neutropenia (S5 and S6 Tables). The quantile range was the range of values between the lowest 25% (25^th^ Percentile) and the highest 25% (75^th^ Percentile) of summary measures or calculated values. The criteria for statistical significance used here was that the p-value from a test is less than or equal to 0.05 (α ≤ 0.05). Multiple group comparisons were made, but no adjustment was made for it. This study is in the early stage of animal research and false positives are preferred to prematurely excluding options for further research.

## Supporting information

S1 Table Pairwise (Exact Wilcoxon Rank Sum) test of total viremia. (DOCX)

S2 Table Testing percent change in absolute lymphocytes and Testing Odds for NOT having lymphopenia. (DOCX)

S3 Table Testing percent change in absolute neutrophils and Testing Odds for NOT having neutropenia. (DOCX)

S4 Table NHP level summary statistics for total viremia. (DOCX)

S5 Table NHP level summary statistics for percent change in absolute lymphocytes and percent change in absolute neutrophils. (DOCX)

S6 Table NHP level summary statistics for days of lymphopenia, mean % lymphopenia, days of neutropenia and mean % neutropenia. (DOCX)

S1 Fig Effect of fluid administration on mice subcutaneously exposed to VEEV. BALB/c mice were exposed to 100 PFU (A & C) or 10,000 PFU (B & D) of VEEV TrD by the subcutaneous route and then administered PBS daily by intraperitoneal route for either 5 or 9 days, as indicated, or left untreated. Average weight (A & B) and survival (C & D) were monitored.
(TIF)
