# Supplementary material for: Therapeutic monoclonal antibody treatment protects nonhuman primates from severe Venezuelan equine encephalitis virus disease after aerosol exposure
Source: PLoS Pathog. 2019 Dec 2;15(12):e1008157. doi: 10.1371/journal.ppat.1008157 (PMC6907853; doi:10.1371/journal.ppat.1008157)
Supplement: S1 Table — (DOCX) [file ppat.1008157.s001.docx]

S1 Table. Pairwise (Exact Wilcoxon Rank Sum) Test of Total Viremia

| **Exp** | **Pairwise Comparison** | **p** | **Median Difference (PFU/mL)** | **95% LCL (PFU/mL)** | **95% UCL (PFU/mL)** |
| --- | --- | --- | --- | --- | --- |
| 1 | 25 mg/kg 1A3B-7 (+1) < Control | 0.04 | -19,000 | -26,200 | 84,100 |
| 2 | 1A3B-7 (+2) < PBS | 0.45 | -250 | -78,100 | 2,000 |
|  | 1A4A-YTE (+1) < PBS | . | 425 | -71,100 | 13,100 |
