# Supplementary material for: Therapeutic monoclonal antibody treatment protects nonhuman primates from severe Venezuelan equine encephalitis virus disease after aerosol exposure
Source: PLoS Pathog. 2019 Dec 2;15(12):e1008157. doi: 10.1371/journal.ppat.1008157 (PMC6907853; doi:10.1371/journal.ppat.1008157)
Supplement: S2 Table — (DOCX) [file ppat.1008157.s002.docx]

S2 Table. Testing Percent Change in Absolute Lymphocytes and Testing Odds for NOT having Lymphopenia

|  | | **Lymphocytes (ABS) Wilcoxon Exact Test (% Change)** | | | | **NOT Lymphopenia Exact Logistic Regression (Odds)** | | | |
| --- | --- | --- | --- | --- | --- | --- | --- | --- | --- |
| **Exp** | **Pairwise Comparison** | **p** | **Median Difference** | **LCL 95%** | **UCL 95%** | **p** | **Odds** | **LCL 95%** | **UCL 95%** |
| 1 | 25 mg/kg 1A3B-7 (+1) > Control | 0.01 | 18.50 | 2.90 | 36.2 | <.01 | 4.50 | 1.610 | 13.60 |
| 2 | 1A3B-7 (+2) > PBS | 0.08 | 11.30 | -3.34 | 28.1 | 0.12 | 2.22 | 0.653 | 8.03 |
|  | 1A4A-YTE (+1) > PBS | 0.27 | 2.13 | -10.40 | 27.1 | 0.07 | 2.63 | 0.760 | 9.90 |
