# Supplementary material for: Therapeutic monoclonal antibody treatment protects nonhuman primates from severe Venezuelan equine encephalitis virus disease after aerosol exposure
Source: PLoS Pathog. 2019 Dec 2;15(12):e1008157. doi: 10.1371/journal.ppat.1008157 (PMC6907853; doi:10.1371/journal.ppat.1008157)
Supplement: S3 Table — (DOCX) [file ppat.1008157.s003.docx]

S3 Table. Testing Percent Change in Absolute Neutrophils and Testing Odds for NOT having Neutropenia

|  | | **Neutrophils (ABS) Wilcoxon Exact Test (% Change)** | | | | **NOT Neutropenia Exact Logistic Regression (Odds)** | | | |
| --- | --- | --- | --- | --- | --- | --- | --- | --- | --- |
| **Exp** | **Pairwise Comparison** | **p** | **Median Difference** | **LCL 95%** | **UCL 95%** | **p** | **Odds** | **LCL 95%** | **UCL 95%** |
| 1 | 25 mg/kg 1A3B-7 (+1) > Control | 0.03 | 37.40 | -2.01 | 76.9 | <.01 | 4.910 | 1.550 | 17.20 |
| 2 | 1A3B-7 (+2) > PBS | 0.50 | 3.41 | -39.80 | 49.6 | 0.17 | 2.030 | 0.580 | 7.47 |
|  | 1A4A-YTE (+1) > PBS | . | -19.00 | -48.80 | 16.8 | . | 0.797 | 0.223 | 2.80 |
