# Supplementary material for: Therapeutic monoclonal antibody treatment protects nonhuman primates from severe Venezuelan equine encephalitis virus disease after aerosol exposure
Source: PLoS Pathog. 2019 Dec 2;15(12):e1008157. doi: 10.1371/journal.ppat.1008157 (PMC6907853; doi:10.1371/journal.ppat.1008157)
Supplement: S4 Table — (DOCX) [file ppat.1008157.s004.docx]

S4 Table. NHP Level Summary Statistics for Total Viremia.

| **Exp** | **Treatment** | **Median (PFU/mL)** | **Quantile Range (PFU/mL)** | **NHPs** | **N** | **Q** |
| --- | --- | --- | --- | --- | --- | --- |
| 1 | 25 mg/kg 1A3B-7 (+1) | 154 | 7,080 | 6 | 46 | 8 |
|  | Control | 23,300 | 7,200 | 6 | 48 | 17 |
| 2 | 1A3B-7 (+2) | 1,230 | 1,180 | 5 | 35 | 10 |
|  | 1A4A-YTE (+1) | 8,250 | 9,290 | 5 | 35 | 14 |
|  | PBS | 1,130 | 3,570 | 5 | 33 | 12 |
| N is the total number of non-missing measurements on all NHPs | | | | | | |
| Q is the total number of quantified measurements on all NHPs | | | | | | |
