# Supplementary material for: Therapeutic monoclonal antibody treatment protects nonhuman primates from severe Venezuelan equine encephalitis virus disease after aerosol exposure
Source: PLoS Pathog. 2019 Dec 2;15(12):e1008157. doi: 10.1371/journal.ppat.1008157 (PMC6907853; doi:10.1371/journal.ppat.1008157)
Supplement: S5 Table — (DOCX) [file ppat.1008157.s005.docx]

S5 Table. NHP Level Summary Statistics for percent change in absolute lymphocytes and percent change in absolute neutrophils.

|  | | | | **Lymphocytes (ABS) in % Change** | | **Neutrophils (ABS) in % Change** | |
| --- | --- | --- | --- | --- | --- | --- | --- |
| **Exp** | **Treatment** | **NHPs** | **N** | **Median** | **Quantile Range** | **Median** | **Quantile Range** |
| 1 | 25 mg/kg 1A3B-7 (+1) | 6 | 40 | -18.40 | 24.60 | 11.30 | 30.00 |
|  | Control | 6 | 39 | -36.60 | 8.39 | -33.90 | 32.80 |
| 2 | 1A3B-7 (+2) | 5 | 35 | -16.20 | 11.30 | -5.63 | 28.40 |
|  | 1A4A-YTE (+1) | 5 | 35 | -23.90 | 3.28 | -25.20 | 25.60 |
|  | PBS | 5 | 34 | -26.60 | 3.70 | -9.04 | 24.30 |
| N is the total number of non-missing measurements on all NHPs | | | | | | | |
