# Supplementary material for: Therapeutic monoclonal antibody treatment protects nonhuman primates from severe Venezuelan equine encephalitis virus disease after aerosol exposure
Source: PLoS Pathog. 2019 Dec 2;15(12):e1008157. doi: 10.1371/journal.ppat.1008157 (PMC6907853; doi:10.1371/journal.ppat.1008157)
Supplement: S6 Table — (DOCX) [file ppat.1008157.s006.docx]

S6 Table. NHP Level Summary Statistics for Days of Lymphopenia, Mean % Lymphopenia, Days of Neutropenia and Mean % Neutropenia

|  | | | | **Lymphopenia in # of Days** | | **Lymphopenia in Mean %** | | **Neutropenia in # of Days** | | **Neutropenia in Mean %** | |
| --- | --- | --- | --- | --- | --- | --- | --- | --- | --- | --- | --- |
| **Exp** | **Treatment** | **NHPs** | **N** | **Mean** | **Standard Deviation** | **Median** | **Quantile Range** | **Mean** | **Standard Deviation** | **Median** | **Quantile Range** |
| 1 | 25 mg/kg 1A3B-7 (+1) | 6 | 40 | 1.8 | 1.470 | 24.3 | 28.6 | 1.5 | 2.070 | 10.0 | 42.9 |
|  | Control | 6 | 39 | 4.2 | 0.753 | 63.3 | 14.3 | 3.5 | 1.520 | 58.6 | 28.6 |
| 2 | 1A3B-7 (+2) | 5 | 35 | 2.0 | 1.220 | 28.6 | 14.3 | 2.4 | 1.520 | 42.9 | 14.3 |
|  | 1A4A-YTE (+1) | 5 | 35 | 1.8 | 1.790 | 28.6 | 42.9 | 3.6 | 2.070 | 57.1 | 14.3 |
|  | PBS | 5 | 34 | 3.0 | 1.410 | 33.3 | 28.6 | 3.2 | 1.300 | 42.9 | 23.8 |
| N is the total number of non-missing measurements on all NHPs | | | | | | | | | | | |
| Maximum Possible Number of Days for Lymphopenia or Neutropenia is Seven (7) | | | | | | | | | | | |
