# Supplementary figures and images for: Therapeutic monoclonal antibody treatment protects nonhuman primates from severe Venezuelan equine encephalitis virus disease after aerosol exposure
Source: PLoS Pathog. 2019 Dec 2;15(12):e1008157. doi: 10.1371/journal.ppat.1008157 (PMC6907853; doi:10.1371/journal.ppat.1008157)

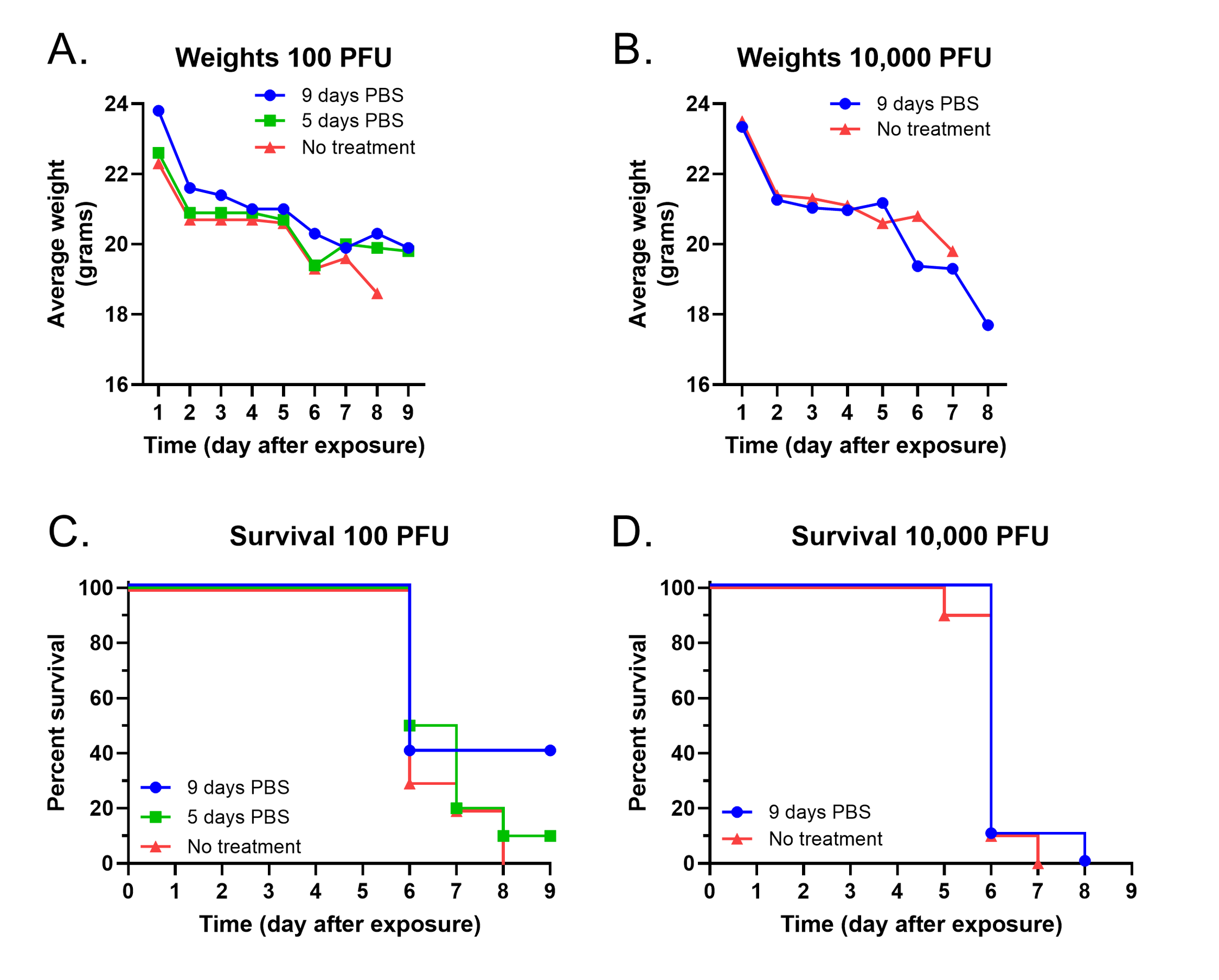

Supplement: S1 Fig — BALB/c mice were exposed to 100 PFU (A & C) or 10,000 PFU (B & D) of VEEV TrD by the subcutaneous route and then administered PBS daily by intraperitoneal route for either 5 or 9 days, as indicated, or left untreated. Average weight (A & B) and survival (C & D) were monitored. (TIF) [file ppat.1008157.s007.tif]
